# Cholecalciferol (vitamin D_3_) has a direct protective activity against interleukin 6-induced atrophy in C2C12 myotubes

**DOI:** 10.18632/aging.202669

**Published:** 2021-02-22

**Authors:** Maraiza Alves Teixeira, Marilisa De Feudis, Simone Reano, Tommaso Raiteri, Andrea Scircoli, Ivan Zaggia, Sara Ruga, Laura Salvadori, Flavia Prodam, Paolo Marzullo, Claudio Molinari, Davide Corà, Nicoletta Filigheddu

**Affiliations:** 1 Department of Translational Medicine, University of Piemonte Orientale, Novara, Italy; 2 Istituto Interuniversitario di Miologia (IIM), Italy; 3 Department of Health Sciences, University of Piemonte Orientale, Novara, Italy; 4 IRCCS Istituto Auxologico Italiano, Laboratory of Metabolic Research, Piancavallo (VB), Italy; 5 Center for Translational Research on Autoimmune and Allergic Disease (CAAD), University of Piemonte Orientale, Novara, Italy

**Keywords:** sarcopenia, cachexia, autophagy, vitamin D hydroxylases, VDR

## Abstract

We previously determined that different vitamin D metabolites can have opposite effects on C2C12 myotubes, depending on the sites of hydroxylation or doses. Specifically, 25(OH)D_3_ (25VD) has an anti-atrophic activity, 1,25(OH)_2_D_3_ induces atrophy, and 24,25(OH)_2_D_3_ is anti-atrophic at low concentrations and atrophic at high concentrations. This study aimed to clarify whether cholecalciferol (VD3) too, the non-hydroxylated upstream metabolite, has a direct effect on muscle cells.

Assessing the effects of VD3 treatment on mouse C2C12 skeletal muscle myotubes undergoing atrophy induced by interleukin 6 (IL6), we demonstrated that VD3 has a protective action, preserving C2C12 myotubes size, likely through promoting the differentiation and fusion of residual myoblasts and by modulating the IL6-induced autophagic flux. The lack, in C2C12 myotubes, of the hydroxylase transforming VD3 in the anti-atrophic 25VD metabolite suggests that VD3 may have a direct biological activity on the skeletal muscle. Furthermore, we found that the protective action of VD3 depended on VDR, implying that VD3 too might bind to and activate VDR. However, despite the formation of VDR-RXR heterodimers, VD3 effects do not depend on RXR activity.

In conclusion, VD3, in addition to its best-known metabolites, may directly impact on skeletal muscle homeostasis.

## INTRODUCTION

Skeletal muscle wasting may occur in several physio-pathological conditions and represents one of the main overlapping features between the physiological age-related sarcopenia and cachexia, which often associates with an underlying disease, such as cancer, especially in terminally ill patients.

In both cases, the occurrence of chronic systemic inflammation may directly contribute to the loss of skeletal muscle mass and functionality [1–5].

Another feature often associated with both muscle wasting-inducing conditions is an important reduction of vitamin D_3_, seen as decreased 25(OH)D_3_ (25-hydroxycholecalciferol, hereafter 25VD) circulating levels [6, 7].

In humans, vitamin D_3_ synthesis begins with skin exposure to ultraviolet B radiation and the consequent photochemical conversion of the precursor, 7-dehydrocholesterol (pro-vitamin D_3_), in pre-vitamin D_3_. Through thermal isomerization, pre-vitamin D_3_ is converted to vitamin D_3_ (cholecalciferol, hereafter VD3), which is hydroxylated in the liver by the 25-hydroxylases (25-OHases encoded by CYP27A1 and CYP2R1 genes); the resultant 25VD is further converted in 1,25(OH)_2_D_3_ (1,25-dihydroxy vitamin D_3_ or calcitriol, hereafter 1,25VD) in the kidney by 1α-hydroxylase (1α-OHase, encoded by the CYP27B1 gene). Lastly, the 24-hydroxylase encoded by the gene CYP24A1 catalyzes the conversion of both 25VD and 1,25VD into 24-hydroxylated products targeted for inactivation and excretion.

1,25VD exerts its biological activities through the binding to vitamin D receptor (VDR), inducing the heterodimerization of VDR with the retinoic acid receptor RXR, the recruitment of various coregulators, and the binding of this complex to specific regions on DNA. Though the bone is the best-known target of vitamin D, VDR is almost ubiquitously expressed through the cells and tissues of the body, suggesting that vitamin D has wide pleiotropic activities. Likewise, the widespread extra-renal expression of the CYP27B1 hydroxylase suggests that 25VD could be locally converted to 1,25VD, thus providing a mechanism explaining the ever-growing observations of 25VD-elicited biological activities [8, 9]. Nevertheless, we have demonstrated that, in C2C12 myotubes, 25VD has a biological anti-atrophic activity that does not depend on its intracellular conversion to 1,25VD. Unexpectedly, in this *in vitro* model of skeletal muscle, 25VD and 1,25VD have antithetical effects, likely *via* differential modulation of 24-hydroxylase [10]. Furthermore, we showed that, on C2C12 myotubes, 24,25(OH)_2_D_3_ (24,25VD) itself could have divergent effects, either atrophic or hypertrophic, depending on its concentration. These observations are in accordance with and strengthen the idea that all vitamin D_3_ metabolites, and not only 1,25VD, might be biologically active [11, 12]. Proceeding with this view, here we explored the hypothesis that VD3 as well could have a direct biological activity on skeletal muscle cells.

## RESULTS

### VD3 treatment protects C2C12 myotubes and primary myofibers from IL6 induced atrophy

**Figure 1:**
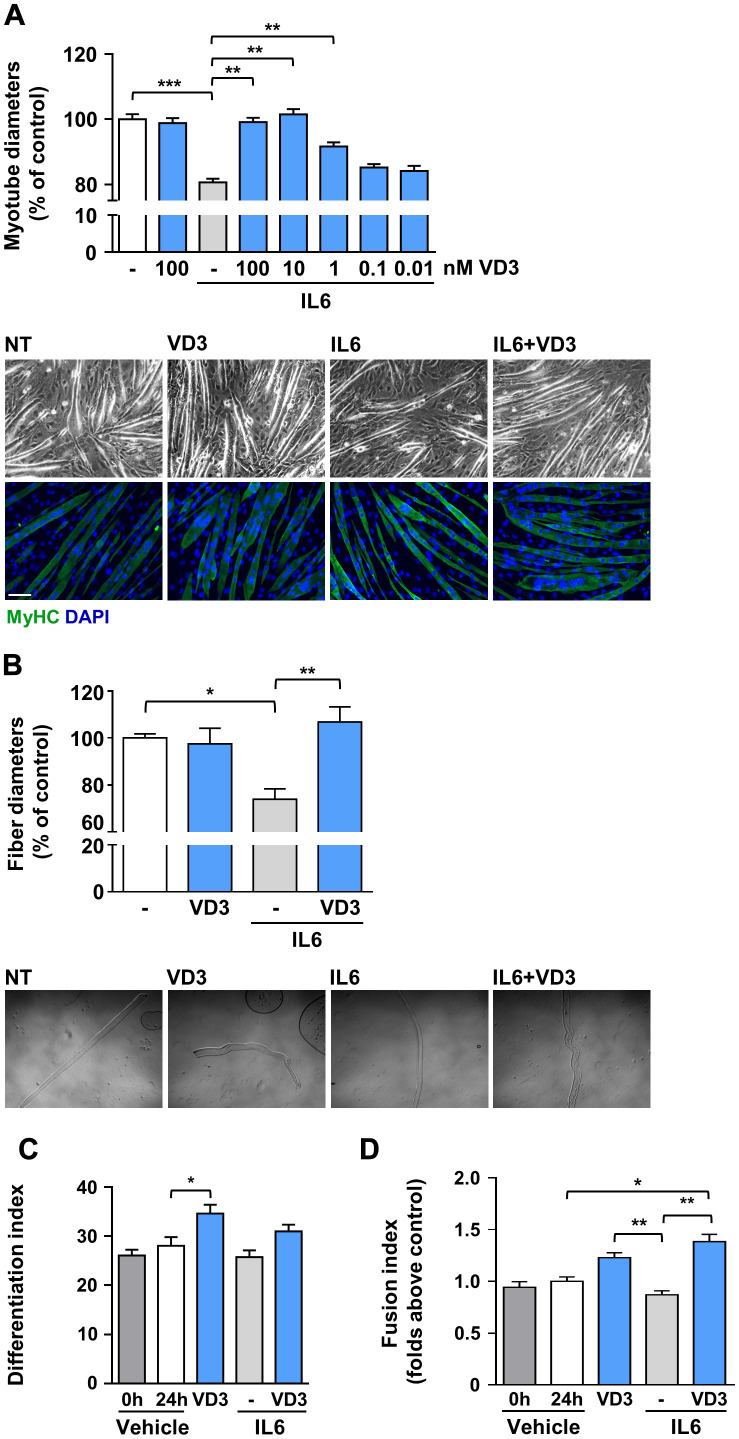
**VD3 protects C2C12 myotubes and isolated myofibers from IL6-induced atrophy.** (**A**) Myotubes were treated in serum-free medium with 100 nM VD3, 20 ng/ml IL6 alone or in combination with decreasing concentrations (100 – 0.01 nM) of VD3 for 24 h. At the end of the indicated treatments, myotube diameters were measured either in phase-contrast images or after immunofluorescence (IF) with anti-MyHC antibody and DAPI counterstaining. Representative images of myotubes in both phase-contrast and IF are shown at the bottom of the panel. Scale bar, 100 μm (**B**) Single myofibers were isolated from C57BL/6 mice and treated with 20 ng/ml IL6 alone or in combination with 100nM VD3. Myofiber diameters were acquired from phase-contrast images (representative images at the bottom of the panel). (**C**) Differentiation and (**D**) fusion indexes after 24 h of VD3 and IL6 treatments compared to the time of treatment administration (0 h; C2C12 differentiated for at least 4 days in DM). Data are presented as the mean ± SEM. *P < 0.05; **P < 0.01; ***P < 0.001.

To assess whether VD3 could have protective activity in the skeletal muscle, we mimicked *in vitro* the condition of inflammation-prompted muscle wasting by treating murine C2C12-derived myotubes with the pro-atrophic cytokine interleukin-6 (IL6) [10]. C2C12 myotubes incubated for 24 h with 20 ng/ml IL6 underwent consistent atrophy, appraised as a reduction of 20% in myotube thickness (Figure 1A). Co-treatment with VD3 prevented the reduction of myotube diameter in a dose-dependent manner, starting from 1 nM concentrations. At concentrations of 10 and 100 nM, VD3 completely blocked the atrophic effect of IL6, while VD3 alone had no evident effects on C2C12 myotube size (Figure 1A). A similar result was obtained treating freshly isolated myofibers with IL6 in the presence or absence of VD3 (Figure 1B), supporting the choice of C2C12 myotubes as a suitable model to study skeletal muscle homeostasis.

To investigate if the changes in myotube diameters upon IL6 and VD3 treatments could derive from differentiative effects on the residual undifferentiated myoblasts in culture [13], we calculated the variation in differentiation and fusion indexes during the experimental period both in control (untreated cultures) and treated conditions. Although there was no additional differentiation of untreated myoblasts cultured in differentiation medium within the experimental period, VD3 promoted a further 20% differentiation that influenced, although not to a statistically significant extent, the differentiation of the combined treatment of IL6 and VD3 (Figure 1C). However, the fusion of residual myoblasts was significantly promoted by VD3 treatment, both alone and in combination with IL6 (Figure 1D). Conversely, IL6 treatment did not affect differentiation or fusion of residual myoblasts.

### VD3 does not promote protein synthesis but activates STAT3

**Figure 2:**
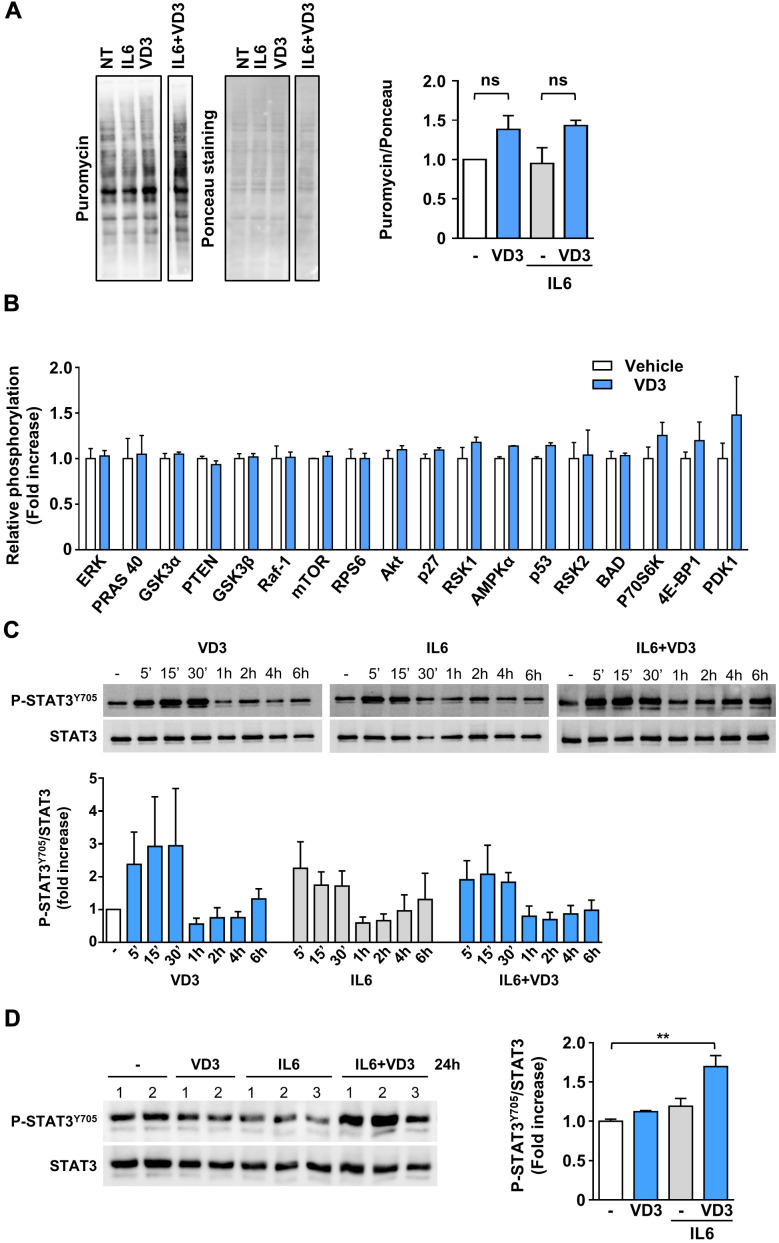
**VD3 does not stimulate protein synthesis but activates STAT3.** (**A**) Protein synthesis upon 24 h of treatment with 20 ng/ml IL6 in the presence or absence of 100nM VD3 was evaluated by SUnSET assay. Incorporation of puromycin into newly synthesized proteins was visualized by Western blotting and normalized on total proteins detected by Ponceau staining (representative images on the left of the panel). Densitometry of three experiments is shown on the right part of the panel. (**B**) Relative phosphorylation of proteins of Akt pathway after 30 min treatment with 100 nM VD3 through protein array analysis. (**C**) Representative western blot (top) and densitometry (bottom) of phosphorylated STAT3^Y705^ levels in C2C12 myotubes treated with 100 nM VD3, 20ng/mL IL6, or their combination for the indicated times. (**D**) Western blot (left) and densitometry (right) of phosphorylated STAT3^Y705^ levels in C2C12 myotubes treated with VD3, IL6, or their combination for 24 h. Data are presented as the mean ± SEM of three independent experiments except for (**D**), in which data are presented as the mean ± SD of the independent experiments indicated on the blot. **P<0.01.

Skeletal muscle homeostasis depends on the balance between anabolic and catabolic processes; however, the lack of any effect of VD3 treatment on myotube and myofiber diameters (Figure 1A, 1B) suggested that VD3 could not stimulate protein synthesis. To verify this hypothesis, we assessed if VD3 could stimulate the *de novo* synthesis of proteins through a SUnSET assay. Although we observed a mild increase in puromycin incorporation (Figure 2A), this was not significant. Coherently, VD3 treatment did not induce the activation of the Akt/mTOR protein network associated with protein synthesis (Figure 2B).

As IL6 promotes muscle wasting mainly through STAT3 activation, and STAT3 inhibition prevents IL6-induced atrophy [14], we investigated if VD3 treatment could curb IL6-elicited STAT3 signaling. As expected, IL6 induced rapid phosphorylation of STAT3^Tyr705^ within the first 30 minutes of treatment. However, unexpectedly, VD3 not only did not inhibit IL6-induced STAT3 activation but induced itself the phosphorylation of STAT3^Tyr705^ (Figure 2C). Even more surprisingly, the co-treatment of VD3 and IL6 induced significant phosphorylation of STAT3^Tyr705^ at 24 h post-treatment that was not induced by the single treatments (Figure 2D).

### VD3 modulates the autophagic flux in IL6-treated C2C12 myotubes

**Figure 3:**
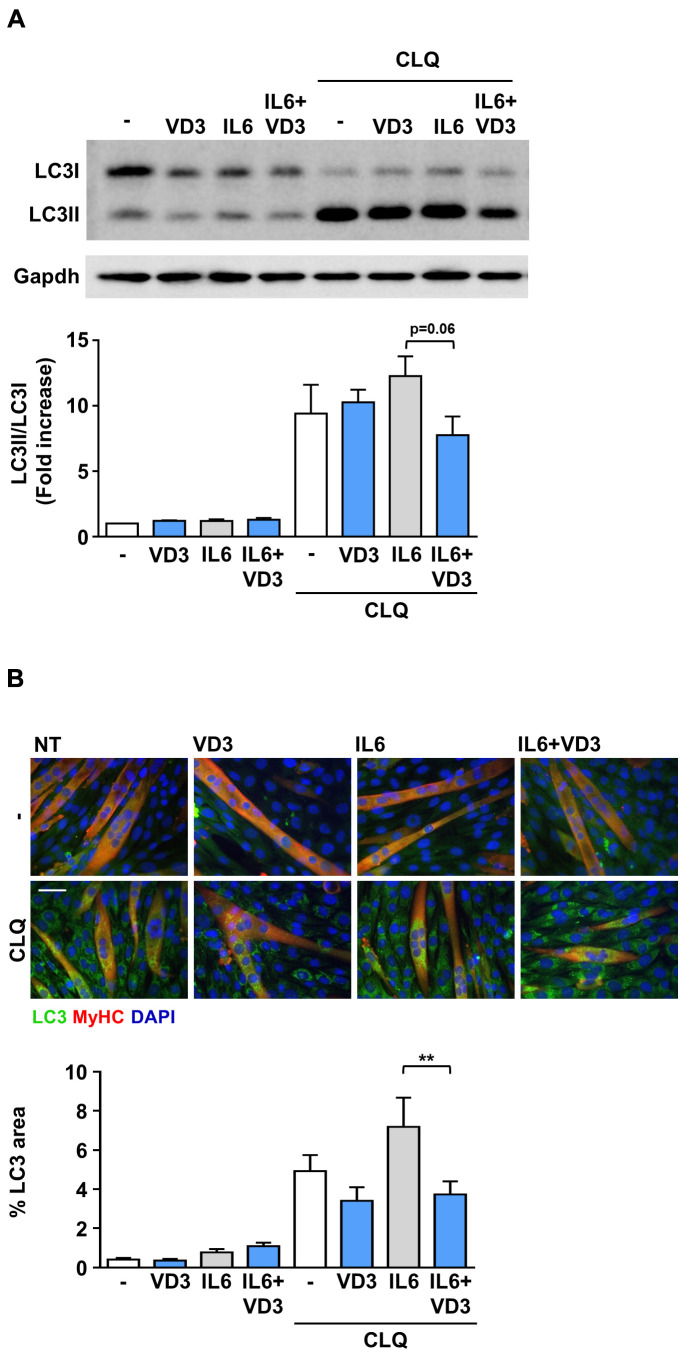
**VD3 modulates the autophagic flux in IL6-treated C2C12 myotubes.** C2C12 myotubes in DM were treated for 24 h with 100 nM VD3, 20ng/mL IL6, or their combination in the presence and absence of 10 μM of chloroquine (CLQ). The autophagosome marker LC3 was detected by western blotting (**A**) or immunofluorescence (**B**). Autophagic flux was assessed by quantifying the ratio of LC3 signal in the presence vs. absence of CLQ. For each panel, representative images are on the top and quantification on the bottom. Scale bar, 50 μm. **P < 0.01.

Autophagy is another mechanism that can regulate muscle homeostasis by regulating protein and organelle turnover [15, 16]. Therefore, we investigated whether IL6, VD3, and their combination could affect autophagy by measuring the accumulation of the lipidated autophagosomal marker LC3II in C2C12 myotubes lysates in the presence or absence of chloroquine, a blocker of the autophagic flux. We observed that the autophagic flux was slightly reduced when VD3 and IL6 were co-administered (Figure 3A). Thus, we assessed the accumulation of LC3 puncta in myotubes by immunofluorescence and observed that the modest increase of IL6-induced autophagic flux was significantly reduced by VD3 co-treatment (Figure 3B), thus confirming the trend perceived in western blot.

### VD3 anti-atrophic activity does not depend on its intracellular conversion to 25VD

**Figure 4:**
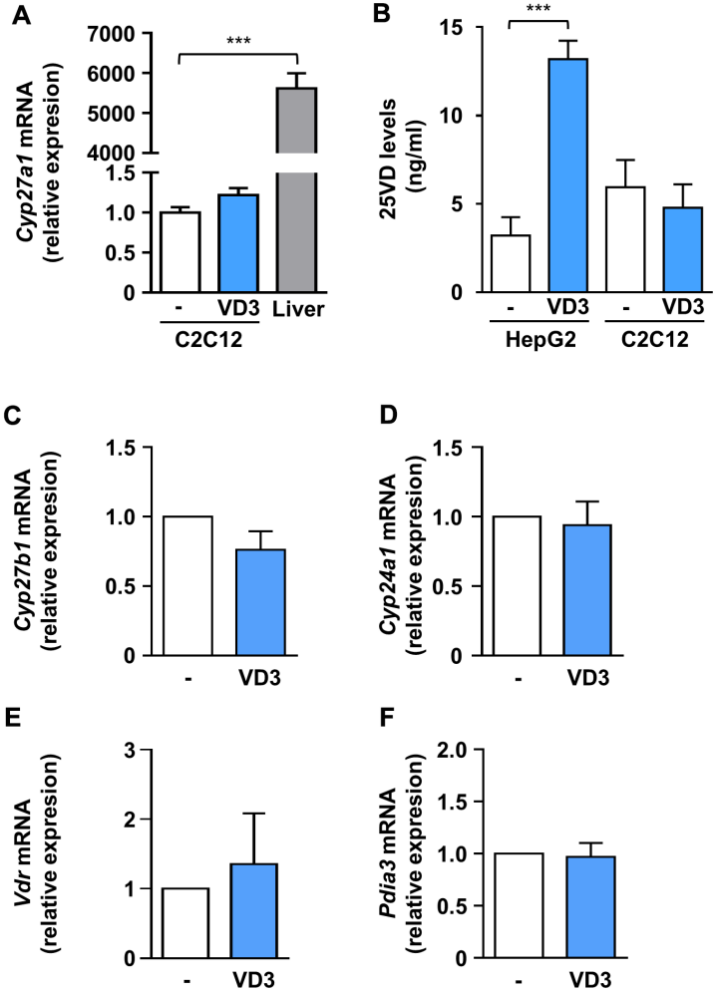
**VD3 did not alter the expression of hydroxylases and receptors involved in vitamin D metabolism and activity, and its anti-atrophic action does not depend on intracellular conversion to 25VD.** (**A**) C2C12 myotubes were treated in serum-free medium with VD3 for 24 h, and mRNA levels of *Cyp27a1* were assayed by real-time PCR, using liver tissue as a positive control and *Gusb* as the housekeeping gene. (**B**) HepG2 cells and C2C12 myotubes were treated in serum-free medium with VD3 for 24 h, and the content of 25VD was quantified in cell lysates with a specific ELISA kit. (**C**–**F**) C2C12 myotubes were treated in serum-free medium as above, and mRNA levels of (**C**) *Cyp27b1*, (**D**) *Cyp24a1*, (**E**) *Vdr*, and (**F**) *Pdia3* were assayed by real-time PCR, using *Gusb* as the housekeeping gene. Data are presented as the mean ± SEM of three independent experiments. ***P < 0.001.

The complexity of vitamin D biological activity is further complicated by the ability of distinct vitamin D metabolites to modulate the expression of the hydroxylases involved in vitamin D metabolism and of VDR [17]. To explore if the hitherto observed effects of VD3 on C2C12 myotubes could depend on its transformation in other metabolites with already proven biological activity, we assessed the expression of the main players of vitamin D metabolism upon VD3 treatment. In particular, as we have previously demonstrated that 25VD has a powerful anti-atrophic activity in C2C12 myotubes treated with pro-cachectic cytokines, including IL6 [10], we explored the hypothesis that the observed anti-atrophic effect of VD3 could be due to its intracellular conversion to 25VD in C2C12 myotubes. *In vivo*, VD3 is hydroxylated in the liver by Cyp2r1 and Cyp27a1 25-hydroxylases to yield 25VD. Using murine liver as a positive control, we found that in C2C12 myotubes *Cyp27a1* showed a low but measurable expression (Figure 4A), while *Cyp2r1* (in mouse, the main 25-hydroxylase [18, 19]) was undetectable, both basally and upon stimulation with VD3 or IL6 (data not shown), suggesting a direct biological action of VD3 on C2C12 myotubes. We verified this hypothesis by quantifying, with a specific ELISA, the 25VD produced intracellularly upon 24 h of VD3 treatment. We demonstrated that C2C12 myotubes, differently from liver-derived cells, were unable to metabolize VD3 into 25VD (Figure 4B), ruling out that the observed anti-atrophic effects derived from the intracellular conversion of VD3 in 25VD.

In addition, we found that, similarly to 25VD and 1,25VD [20], VD3 did not affect the expression of *Cyp27b1*, coding for the 1α-hydroxylase (Figure 3C). However, differently from 25VD and 1,25VD metabolites [10, 20], VD3 did not modulate the expression of the 24-hydroxylase gene *Cyp24a1* (Figure 4D), nor that of *Vdr* (Figure 4E). Also, VD3 did not affect the expression of *Pdia3*, the gene encoding for the 1,25D3-MARRS (Membrane Associated, Rapid Response Steroid binding), an alternative vitamin D receptor regarded as the one mediating the membrane-initiated signaling of 1,25VD (Figure 4F).

### VD3 anti-atrophic activity is VDR-dependent but RXR-independent

**Figure 5:**
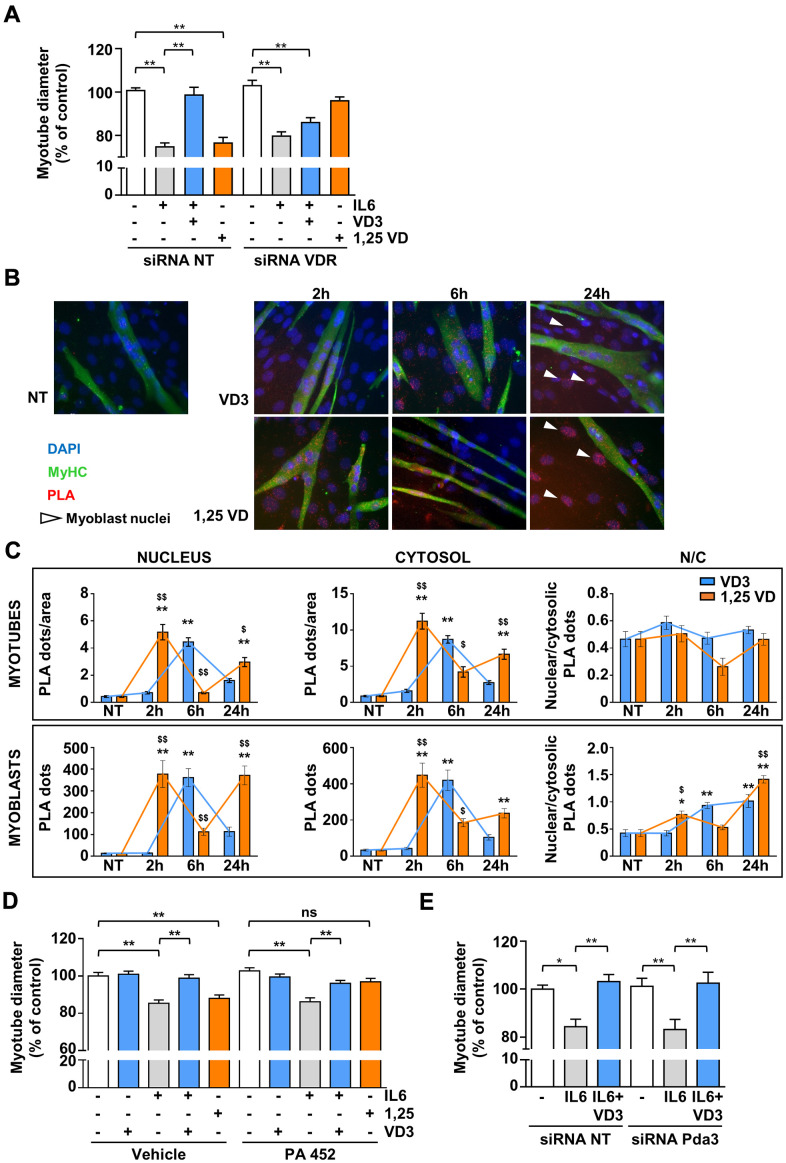
**VDR, but not RXR nor 1,25D3-MARRS (Pdia3), mediates VD3 anti-atrophic activity.** (**A**) C2C12 myotubes were transfected with non-targeting or *Vdr* specific siRNAs. 24 h after silencing, C2C12 myotubes were treated with 20ng/mL IL6 in the absence or presence of 100 nM VD3 or with 100 nM 1,25VD, and myotube diameters were measured after further 24 h. (**B**) Representative images of the PLA-detected complexes of VDR and RXR (red) in C2C12 myotubes (green) treated for the indicated times with 100 nM VD3 or 100 nM 1,25VD. Nuclei were stained with DAPI. The white arrows indicate the nuclei of *bone fide* residual undifferentiated myoblasts. (**C**) Quantification of the PLA dots in myotubes (top) or myoblasts (bottom), in nuclei (left), cytoplasm (middle), and their ratio (right). (**D**) Myotube diameters of C2C12 treated with 20ng/mL IL6, with or without 100 nM VD3, or with 100 nM 1,25VD, in the presence/absence of 2.5 μM RXR inhibitor PA 452. (**E**) C2C12 myotubes were transfected with non-targeting or *Pdia3* specific siRNAs, treated with 20ng/mL IL6 in the absence or presence of 100 nM VD3 and analyzed after further 24 h. Data are presented as the mean ± SEM. *P < 0.05; **P < 0.01; ***P < 0.001 vs. non-treated controls (NT); ^$^*P* < 0.05; ^$$^*P* < 0.01 vs. VD3-treated cells.

Although VD3 does not modulate the expression of *Vdr* (Figure 4E), the silencing of *Vdr* completely abrogated the protective activity of VD3 against IL6-induced atrophy (Figure 5A), indicating that VDR mediates VD3 anti-atrophic activity in C2C12 myotubes. The atrophic activity of 1,25VD [10] was abolished by VDR silencing, as expected. Through a proximity ligation assay (PLA), we observed that, in C2C12 myotubes, VD3, similarly to 1,25VD, induced the formation of VDR-RXR complexes, although with different kinetics (Figure 5B, 5C). Notably, the formation of the VDR-RXR complexes was evenly distributed in both cytoplasmic and nuclear compartments, without a remarkable nuclear translocation, *i.e.*, an increase in nuclear/cytoplasmic ratio (upper panels of Figure 5C). Conversely, in the residual myoblasts (visible in the representative pictures as nuclei belonging to myosin heavy chain (MyHC)-negative cells), both VD3 and 1,25VD promoted both the formation of VDR-RXR complexes over time and their nuclear translocation (lower panels of Figure 5C).

However, despite the formation of VDR-RXR complexes, differently from 1,25VD, RXR activity was not required for the effects of VD3 in C2C12 myotubes, as the inhibition of RXR with 2.5 μM PA 452 abolished only 1,25VD but not VD3 effects (Figure 5D). We finally assessed the involvement of the alternative 1,25D3-MARRS receptor in VD3 anti-atrophic action. The silencing of *Pdia3* did not affect the activity of VD3 (Figure 5E), indicating that 1,25D3-MARRS does not mediate VD3 anti-atrophic effect.

### VD3 does not protect C2C12 myotubes from dexamethasone-induced atrophy

**Figure 6:**
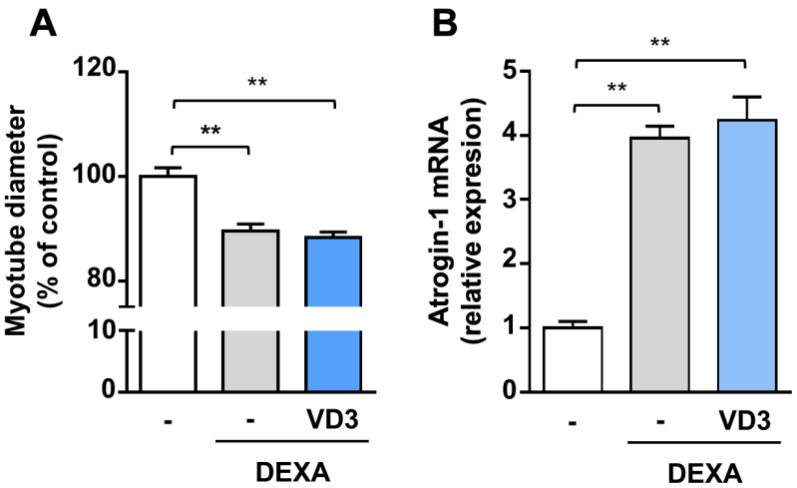
**VD3 does not protect myotubes against atrophy induced by dexamethasone.** (**A**) Myotube diameters were measured after 24 h treatment with 5 μM dexamethasone (DEXA) alone or in combination with 100 nM VD3. (**B**) Expression of Atrogin-1 (*Fbxo32*) was assessed by real-time RT-PCR. Data are presented as the mean ± SEM. ** *P* <0.01.

To investigate if VD3 could have a broad anti-atrophic activity, we assessed its effect by co-treating C2C12 myotubes undergoing atrophy upon treatment with dexamethasone, a synthetic glucocorticoid that induces muscle protein degradation *via* the activation of the ubiquitin-proteasome system [21]. Myotubes were treated with 5 μM dexamethasone for 24 h in the presence or absence of 100 nM VD3. Treatment with dexamethasone reduced myotube diameters by 10% and induced the muscle-specific ubiquitin ligase Atrogin-1 (MAFbx) expression. VD3 was not able to inhibit this atrophy (Figure 6A, 6B), indicating that VD3 ability to counteract muscle atrophy is limited.

## DISCUSSION

The impact of vitamin D on muscle morphology and functionality has been largely investigated, and vitamin D deficiency has been implicated in pathological conditions such as frailty and sarcopenia in the elders and cachexia in cancer patients [6, 22]. Even though vitamin D deficiency correlates with muscular impairment, the effectiveness of vitamin D supplementation depends on the context of use. In fact, a relevant number of epidemiological studies have suggested the potential role of vitamin D intake to maintain or improve muscle strength and function in older people [23]. On the other hand, the efficacy of vitamin D supplementation in cancer cachexia associated with low levels of plasmatic vitamin D remains non-conclusive, both in human and in rodent experimental models [22, 24, 25].

In our previous attempt to better understand these different biological responses in the skeletal muscle to vitamin D supplementation, we realized that, *in vitro*, different vitamin D metabolites have distinctive activities. In particular, we conjectured that a different extent of 24-hydroxylase modulation by 25VD and 1,25VD results in divergent effects on C2C12 myotubes, being 25VD protective against cytokine-induced atrophy and 1,25VD atrophic *per se*. Furthermore, 24,25VD itself can either protect or induce atrophy, depending on its concentration [10].

Here, we showed that the un-hydroxylated VD3 could prevent IL6-induced atrophy in C2C12 myotubes and primary myofibers (Figure 1). Notably, the absence in these cells of *Cyp2r1*, the principal vitamin D 25-hydroxylase in mice [18, 19], together with the inability of the other vitamin D 25-hydroxylase, *Cyp27a1*, to convert VD3 in 25VD in C2C12 myotubes (Figure 4B), rules out an intracellular conversion of VD3 in 25VD, whose anti-atrophic activity has already been demonstrated [10] and implies a direct activity of VD3 in C2C12 myotubes. In addition, the loss of anti-atrophic activity upon silencing of *Vdr* (Figure 4A) suggests that VDR could be bound by VD3 as well, besides 1,25VD and 25VD [26], although specific binding studies should be performed to confirm this hypothesis and determine the binding affinity. Furthermore, similarly to 1,25VD, VD3 induced the formation of VDR-RXR complexes, although with different kinetics (Figure 5B, 5C). Contrary to what one might expect, 1,25VD did not induce the nuclear translocation of VDR-RXR complexes in myotubes (Figure 5C). It should be noted, however, that the 1,25VD-induced increase of the cellular amount of VDR [24] could partially disguise the monitoring of VDR-RXR translocation. The fact that RXR activity is dispensable for VD3 anti-atrophic action (Figure 5D) might suggest that the VD3-induced VDR-RXR interaction is less stable than that induced by 1,25VD. However, the hypothesis that VD3 binding to VDR does not stabilize the VDR-RXR complex needs further investigation. Intriguingly, VDR-RXR complexes showed a different pattern of cytosolic/nuclear localization in C2C12 myotubes vs. myoblasts (Figure 5B, 5C). Indeed, VDR-RXR complexes were evenly distributed in myotubes after VD3 or 1,25VD stimulation, while in myoblasts, both VD3 and 1,25VD clearly induced an increase of the VDR-RXR signal in the nuclei (Figure 5C). Although 1,25VD induces the increase of VDR in myoblasts as well, we hypothesize that the higher amount of VDR in myoblasts vs. myotubes [24] made it easier to visualize the nuclear translocation in this case. Altogether, these findings could account for the sometimes-discordant data in the literature about the effect of vitamin D metabolites in C2C12 or muscle-derived cells of different origin, as some studies were carried out on myoblasts [27–30], others during the differentiation from myoblasts to myotubes [29–31], and yet some others in differentiated myotubes [10, 24]. The presence of VDR-RXR complexes in nuclei upon VD3 treatment might imply that the anti-atrophic effect is genomic. Also, the lack of involvement of the alternative vitamin D receptor considered the one mediating the non-genomic activity of 1,25VD (Figure 5E) tends in that direction, although this notion should be further assessed with appropriate methods.

Our data indicate that the anti-atrophic activity of VD3 is not generic but depends on the stimulus inducing atrophy. Indeed, VD3 was unable to protect from dexamethasone-induced atrophy and to prevent the induction of the muscle-specific ubiquitin ligase Atrogin-1 (Figure 6). We could not detect an increase in Atrogin-1 expression in C2C12 myotubes upon IL6 treatment (data not shown), and indeed the ability of IL6 to induce Atrogin-1 remains debated, with several works reporting contrasting findings [32]. Therefore, we can speculate that VD3 can protect from stimuli that do not involve, as the primary mechanism, the induction of atrogenes (muscle-specific ubiquitin ligases) and activation of the proteasome system. Another reason for the lack of protection against dexamethasone-induced atrophy might lie in the heterodimerization of the glucocorticoid receptor (GR) with RXR, the main partner for VDR heterodimerization upon ligand binding. GR-RXR dimerization occurs in the case of dexamethasone-induced death of murine T-cells and thymocytes [33]. In the light of the involvement of VDR in the anti-atrophic activity of VD3 (Figure 5A), it is possible to speculate that dexamethasone signaling could induce the dimerization of GR with RXR, thus preventing the formation of VDR-RXR complex and the anti-atrophic activity of VD3. However, RXR activity is not required for VD3 anti-atrophic activity (Figure 5D); therefore, it is possible that the strong GR activation induced by dexamethasone could trap coregulators like steroid receptor coactivator-1 (SRC-1), modifying the mutual binding to glucocorticoid- and vitamin D-responsive elements (GREs and VDREs, respectively) on DNA and, consequently, the activation/inhibition of specific genes [34–36].

On the other hand, IL6-induced muscle atrophy mainly depends on the activation of STAT3, as its inhibition prevents muscle wasting [14]. Contrary to what one could expect, VD3, instead of inhibiting STAT3 activation, phosphorylated it *per se* (Figure 2C). However, STAT3 activation in skeletal muscle has pleiotropic functions and apparently contradictory effects. In particular, STAT3 has a pivotal role in satellite cell myogenic progression *in vitro* and *in vivo* [37, 38]. Notably, treatment of myoblasts with sub-atrophic doses of IL6 induced a different timing of JAK2/STAT3 signaling pathway activation depending on the concentrations used that, eventually, promoted either cell proliferation or differentiation [37]. We can speculate that a fine-tuning of STAT3 activation may lead C2C12 myotubes to different fates and that VD3-induced activation of STAT3, either alone or in combination with IL6, tips the scales in favor of residual myoblasts differentiation instead of atrophy. A similar mechanism could also explain the effects on autophagy, as STAT3 activation may affect autophagy, with effects either anti- or pro-autophagic, depending on the cells and stimuli [39]. In skeletal muscle, STAT3 activation inhibits starvation-induced macroautophagy, especially in glycolytic fibers [40, 41]. In C2C12 myotubes, despite both IL6 and VD3 induced rapid STAT3 phosphorylation, only IL6 seemed to promote a modest autophagy, while co-treatment with VD3 clearly reduced it (Figure 3B). Notably, at 24 h post-treatment, STAT3 was phosphorylated only upon VD3 and IL6 co-treatment (Figure 2D). If the effect of VD3 on autophagy induction depends on the late reactivation of STAT3 still needs to be investigated. Nevertheless, contrary to 25VD [10], the modulation of autophagy does not seem to be a key mechanism underlying the anti-atrophic activity of VD3.

Collectively, our findings suggest that VD3 supplementation *in vivo* (where it is physiologically converted in 25VD, 1,25VD, and 24-hydroxylated products) can result in a combination of effects due to the simultaneous action of different vitamin D metabolites, each of which with its own pro- or anti-atrophic activity [10]. Moreover, although C2C12 myotubes do not express *Cyp2r1*, this 25-hydroxylase is present in the whole muscle [42, 43], thus possibly transforming VD3 in 25VD. Although VD3 did not modulate the expression of the other hydroxylases involved in vitamin D metabolism (Figure 4), some of those may be modulated by other vitamin D metabolites [10, 20] and by systemic inflammation [44] in a tissue-specific manner, further raveling the scenario and providing a putative explanation for the contrasting outcomes of VD3 supplementation in different conditions. In conclusion, our findings suggest the need to assess, before VD3 supplementation *in vivo*, the levels of as many as possible vitamin D metabolites as a readout of the activity of different hydroxylases, thus avoiding the increase of the pro-atrophic metabolites. Also, the design of VD3-derived not-hydroxylable molecules that maintain the anti-atrophic property of VD3 preventing the sequential hydroxylations that could result in pro-atrophic molecules might represent a prominent strategy to overcome the limitations of VD3 supplementation in some pathological conditions.

## MATERIALS AND METHODS

### Reagents

VD3 and 1,25VD were purchased from Cayman Chemicals (Ann Arbor, Michigan, USA) and dissolved in ethanol. IL-6 was from Peprotech (London, UK). Water-soluble dexamethasone was from Merck Life Science (Milan, Italy). The RXR antagonist PA 452 was from Tocris Bioscience (Bristol, UK), the anti-LC3 antibody was from Proteintech (Rosemont, Illinois, USA), the anti-STAT3 and anti-phospho-STAT3^Tyr705^ antibodies were from Cell Signaling Technology (Beverly, MA), and the anti-puromycin antibody was from Merck Life Science. The ELISA kit for 25VD quantification was from Enzo Life Sciences (Lausen, Switzerland). All other reagents, unless otherwise stated, were from Merck Life Science.

### Cell cultures and myotube analysis

C2C12 myoblasts were grown at low density in DMEM (Gibco, Thermo Fisher Scientific, Waltham, Massachusetts, USA) supplemented with 10% fetal bovine serum (FBS, Gibco, Thermo Fisher Scientific), 100 U/mL penicillin, 100 μg/mL streptomycin, and 0.25 μg/mL antimycotic in a humidified 5% CO2 incubator at 37° C. To induce differentiation, cells were allowed to become confluent, and the medium was switched to differentiation medium (DM), consisting in DMEM supplemented with 2% horse serum (GE Healthcare Bio-Sciences, Uppsala, Sweden), penicillin, streptomycin, and antimycotic as described above. Unless otherwise specified, myotubes were treated after at least 4 days of differentiation in serum-free medium. Control cells were treated with ethanol. Myotube diameters were measured with ImageJ software as previously described [45].

HepG2 (human hepatoma) cells were cultured in DMEM supplemented with 10% FBS and penicillin, streptomycin, and antimycotic as above. Before treatments, cells were cultured overnight in serum-free medium.

### Differentiation index and fusion index

Differentiation and fusion indexes were calculated as number of myosin heavy chain (MyHC)-positive cells above the total number of nuclei and average number of nuclei in myotubes with at least 3 nuclei above the total nuclei, respectively, as previously described [46].

### Myofiber isolation and culture

Single myofibers were isolated as previously described [47]. Briefly, EDL muscles from C57BL/6 mice were digested in 0.2% collagenase type-I in DMEM for 60–70 minutes at 37° C. Muscles were mechanically dissociated, and single fibers liberated. After extensive washing, myofibers were cultured in low proliferation medium (LPM, DMEM supplemented with 10% HS and 0.5% CEE) in suspension. Treatments were added in LPM immediately after fiber seeding, and 24 h later fibers were fixed in 4% PFA for 10 minutes. Myofiber microphotographs (10 per treatment, 10X magnification) were acquired with a bright field microscopy (Zeiss), and fiber diameters were measured with ImageJ software.

### Western blotting

At the end of the indicated treatments, cells were washed in ice-cold PBS and solubilized with a lysis buffer containing 1% Triton X-100, 0.1% sodium deoxycholate, 0.1% sodium dodecyl sulfate, 1 mM EDTA, 1 mM EGTA, 50 mM NaF, 160 mM NaCl, 20mM Tris-HCl, pH 7.4, and supplemented with protease inhibitor cocktail. Lysates were stirred at 4° C for 15 minutes and centrifuged at 15 000 g for 15 minutes at 4° C. Protein concentration was determined by BCA protein assay kit (Thermo Fisher Scientific). Proteins (10-20 μg protein/lane) were separated by 10% or 15% SDS-PAGE and transferred to polyvinylidene difluoride filters (PVDF) (Hybond-P; GE Healthcare, Little Chalfont, Buckinghamshire, UK). Membranes were saturated with 4% bovine serum albumin (BSA), incubated with the primary antibodies (1:1000) overnight, washed with Tris buffered saline (TBS) 0.1% Tween, incubated with the appropriate secondary antibody (1:3000; Invitrogen, Thermo Fisher Scientific), visualized with Western Lightning Chemiluminescence Reagent Plus (PerkinElmer Life and Analytical Sciences, Waltham, Massachusetts, USA), acquired with ChemiDoc Touch (Bio-Rad, Hercules, California, USA), and analyzed with ImageLab (Bio-Rad). After anti-phospho-STAT3^Tyr705^, membranes were stripped with Restore Plus Western blot stripping buffer (Thermo Fisher Scientific) and reblotted with the corresponding total protein antibody.

### SUnSET protein synthesis assay

Protein synthesis was evaluated by SUnSET assay [48]. Differentiated myotubes were treated with 100 nM VD3 in serum-free medium for 24 h, and, 30 minutes before cell lysis, 500 ng/mL puromycin was added to the medium. After western blotting with an anti-puromycin antibody, membranes were stained with Ponceau S red to visualize total proteins. Both western blots and stained membranes were acquired with ChemiDoc Touch (Bio-Rad, Hercules, California, USA), and analyzed with ImageLab (Bio-Rad).

### AKT pathway phosphorylation array

Cells were solubilized as for western blotting, and mouse AKT pathway phosphorylation arrays C1 (RayBiotech, Inc, Norcross, Georgia, USA) were used according to manufacturer instructions.

### Intracellular quantification of 25VD

C2C12 myotubes and HepG2 cells were treated for 24 h with 100 nM VD3. At the end of the treatment, cells were washed in ice-cold PBS and solubilized with a lysis buffer containing 1% Triton X-100, 0.5% sodium deoxycholate, 1 mM EDTA, 1 mM EGTA, 150 mM NaCl, 100 mM Tris-HCl, pH 7.4. Lysates were stirred at 4° C for 15 min and centrifuged at 15 000 g for 15 minutes at 4° C. The amount of 25VD produced by the cells was quantified using the *25(OH) Vitamin D ELISA Kit* (Enzo Life Sciences) according to manufacturer instructions.

### Immunofluorescence

For differentiation and fusion index quantification, myotubes were fixed in cold methanol:acetone 1:1 for 10 minutes, permeabilized with 0.2% triton for 5 minutes and blocked with 4% BSA for 30 minutes. Primary antibody to detect MyHC (1:10; Developmental Studies Hybridoma Bank, Iowa City, IA) was incubated overnight at 4° C, and the secondary antibody (Alexa Fluor Dyes-conjugated 488 anti-rabbit 1:400; Thermo Fisher Scientific) for 1 h at RT, followed by 5 minutes of DAPI. Images were acquired with an EVOS™ XL Core Imaging System (ThermoFisher Scientific) and quantification was performed with ImageJ.

For LC3 detection, C2C12 myotubes were fixed in 4% paraformaldehyde for 10 minutes, washed with PBS, permeabilized with HEPES-TRITON X-100 0.5% for 5 minutes, and washed two times with PBS-0.2% BSA. For blocking the unspecific binding sites, cells were incubated in 4% BSA for 1 h at RT. LC3-positive puncta were evaluated by incubation for 1 h with rabbit anti-LC3 (1:100), followed by the appropriate Alexa Fluor Dye-conjugated secondary antibody (488 anti-rabbit 1:400; Thermo Fisher Scientific). Nuclei were counterstained with DAPI (1:100, Thermo Fisher Scientific). Images were acquired with a Leica dm 5500b fluorescence microscope (Leica, Wetzlar, Germany) equipped with Leica Application Suite X software, using a 40X objective, and fluorescence signals, normalized to the area of myotubes, were quantified with ImageJ software.

### Proximity ligation assay (PLA)

For the PLA (Duolink, Merck Life Science), the manufacturer instructions were slightly modified to allow the identification of myoblasts and myotubes. In brief, C2C12 cells were plated and differentiated on glass coverslips. At the end of the indicated treatments, myotubes were rinsed three times with PBS and fixed in 4% PFA in PBS for 10 minutes. The cells were permeabilized in 0.2% Triton X-100 for 5 minutes and blocked with 4% BSA in PBS for 60 minutes at 37° C. After blocking, cells were incubated with antibodies against VDR (1:50, Santa Cruz Biotechnology, Santa Cruz, CA), RXR (1:50, Proteintech, Rosemont, Illinois, USA), and MyHC (1:10) in PBS overnight at 4° C, followed by incubation with the corresponding secondary antibodies conjugated with PLA probes for 60 minutes at 37° C in the dark. Cells were washed three times in PBS. Duolink and DAPI signals were detected using the 40X objective on the Leica dm 5500b fluorescence microscope.

### RNA extraction and analysis

Total RNA from myotubes was extracted by RNAzol (SigmaAldrich). The RNA was retro-transcribed with High-capacity cDNA Reverse Transcription Kit (Applied Biosystems, Thermo Fisher Scientific) and real-time PCR was performed with the StepOnePlus Real-time PCR System (Applied Biosystems, Thermo Fisher Scientific), using the following TaqMan probes (Thermo Fisher Scientific): Mm00499518_ m1 (*Fbxo32*, Atrogin-1/MAFbx), Mm00487244_m1 (*Cyp24a1*), Mm01165918_g1 (*Cyp27b1*), Mm00470430_m1 (*Cyp27a1*), Mm00437297_m1 (*Vdr*), Mm00433130_m1 (*Pdia3*), Mm01159413_m1 (*Cyp2r1),* Mm01197698_m1 (*Gusb*).

### VDR and PDIA3 silencing

A concentration of 10 nM of *Vdr* siRNA, *Pdia3* siRNA (Integrated DNA Technologies), or siRNA negative control sequence (Integrated DNA Technologies) was transfected with Lipofectamine 3000 (Invitrogen, Thermo Fisher Scientific) in C2C12 at day 3 of differentiation. Myotubes underwent treatments with IL6 and VD3 24 h later. Transfection of myotubes with Block-iT (Invitrogen, Thermo Fisher Scientific) was used to assess transfection efficiency, which was always >90%.

### Data analysis

The investigators quantifying the experimental outcomes were maintained blinded to the treatments and the statistic evaluation of the experimental data was performed by another investigator not directly involved in data collection and parameters measurement.

Unless otherwise specified, at least triplicate samples from three independent experiments were analyzed. Data are presented as the mean ± SEM. Outliers in the measurements were identified by mean of the interquartile range (IQR), as either below Q1 - 1.5 IQR or above Q3 + 1.5 IQR, and excluded from the analysis. The variation among groups was evaluated using ANOVA test followed by Tukey’s Multiple comparisons test unless otherwise stated. Statistical significance was assumed for P < 0.05. All statistical analyses were performed with GraphPad Prism 6.
